# Artificial intelligence-based conversational agent to support medication prescribing

**DOI:** 10.1093/jamiaopen/ooaa009

**Published:** 2020-05-01

**Authors:** Anita M Preininger, Brett South, Jeff Heiland, Adam Buchold, Mya Baca, Suwei Wang, Rex Nipper, Nawshin Kutub, Bryan Bohanan, Gretchen Purcell Jackson

**Affiliations:** o1 IBM Watson Health, Cambridge, MA, USA; o2 Department of Surgery, Vanderbilt University Medical Center, Nashville, TN, USA; o3 Department of Biomedical Informatics, Vanderbilt University Medical Center, Nashville, TN, USA; o4 Department of Pediatrics, Nashville, TN, USA

**Keywords:** conversational agents, pharmacological information systems, machine learning, natural language processing, artificial intelligence

## Abstract

**Objective:**

This article describes the system architecture, training, initial use, and performance of Watson Assistant (WA), an artificial intelligence-based conversational agent, accessible within Micromedex^®^.

**Materials and methods:**

The number and frequency of *intents* (target of a user’s query) triggered in WA during its initial use were examined; intents triggered over 9 months were compared to the frequency of topics accessed via keyword search of Micromedex. Accuracy of WA intents assigned to 400 queries was compared to assignments by 2 independent subject matter experts (SMEs), with inter-rater reliability measured by Cohen’s kappa.

**Results:**

In over 126 000 conversations with WA, intents most frequently triggered involved dosing (*N* = 30 239, 23.9%) and administration (*N* = 14 520, 11.5%). SMEs with substantial inter-rater agreement (kappa = 0.71) agreed with intent mapping in 247 of 400 queries (62%), including 16 queries related to content that WA and SMEs agreed was unavailable in WA. SMEs found 57 (14%) of 400 queries incorrectly mapped by WA; 112 (28%) queries unanswerable by WA included queries that were either ambiguous, contained unrecognized typographical errors, or addressed topics unavailable to WA. Of the queries answerable by WA (288), SMEs determined 231 (80%) were correctly linked to an intent.

**Discussion:**

A conversational agent successfully linked most queries to intents in Micromedex. Ongoing system training seeks to widen the scope of WA and improve matching capabilities.

**Conclusion:**

WA enabled Micromedex users to obtain answers to many medication-related questions using natural language, with the conversational agent facilitating mapping to a broader distribution of topics than standard keyword searches.

## INTRODUCTION

The prevalence of preventable adverse drug events (ADEs) in hospital populations ranges from 0.6 per 100 hospitalized patients^1^ to as many as 1 in 10,^2^ with related costs ranging from $6 to $29 billion annually.^2^ Pharmacological information systems can improve access to drug information and potentially decrease ADEs by automating content retrieval and providing evidence-based information.^3–6^ Micromedex^®^ is a pharmacological knowledge base supported by evidence from current literature and resources.^6^ The core content in Micromedex is developed through curation of pharmacological, regulatory, biomedical, and scientific information by pharmacy specialists, medical librarians, and biostatisticians. Content is evaluated for clinical significance and accuracy by experts in drugs, diseases, toxicology, and patient education, with additional review of critical content areas by editorial board members, outside peer reviewers, academic scientists, and healthcare professionals.

Micromedex is used globally by roughly 4500 healthcare organizations and is a key resource for Poison Control Centers^7^ as well as Medicare and Medicaid^8^ evaluation of off-label drugs in the United States. Micromedex contains a comprehensive listing of drug–drug interactions^9–13^; many commonly used drug combinations are available for review in Micromedex.^9–11^^,^^14–16^ Micromedex details information on the frequency, severity, and management of drug reactions.^17^ It uniquely offers users side-by-side comparisons of drug monographs,^10^ as well as natural language search capability through Watson Assistant (WA).

WA is an artificial intelligence (AI)-based conversational agent powered by Watson, a supercomputer that relies on IBM’s DeepQA to support advanced analytics and information retrieval. WA, integrated with Micromedex, enables users to ask medication-related questions using natural language. With natural language processing (NLP) and machine learning (ML), WA functions as a pharmacological question-answering system.^18^^,^^19^

## OBJECTIVE

The objective of this article is to describe the architecture of the conversational agent, WA, to describe an initial experience with system use, detailing the types of queries entered and to evaluate system success in mapping queries to appropriate content.

### System overview

**Figure 1.**
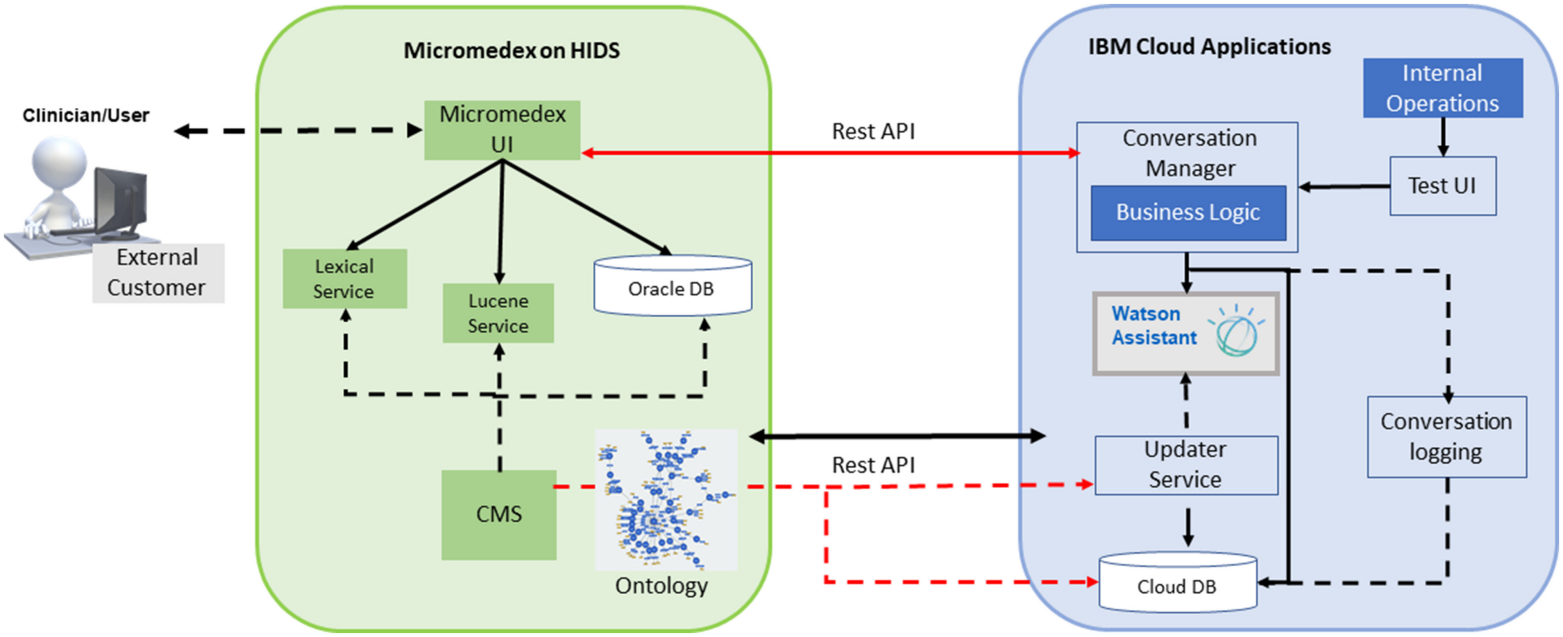
System architecture. CMS, content management system; DB, database; HIDS, health internet delivery system; UI, user interface. Dotted lines, data flow; red lines, representational state transfer application (REST) services; solid lines, data retrieval.

The system architecture for Micromedex equipped with WA is depicted in Figure 1. The combined health internet delivery system (HIDS) consists of components that are proprietary to the Micromedex user interface (UI, Figure 1, green), including tools to disambiguate lexical variants, acronyms and abbreviations, a Lucene service, Oracle database (DB), a content management system (CMS), and ontology. Keyword search of Micromedex content is mediated by the open-source Lucene service. The lexical service integrates clinical terminology and taxonomies, helping to connect keyword entries with clinical terminology. The CMS contains drug content which is created and maintained by clinical editors; drug content maintained in the Oracle database originates from the CMS. The Oracle database contains Micromedex’s Quick Answers, housing summary-level drug information accessed by cloud database 2 (DB2). The ontology is a representation of summary-level information, defined by organization of drug information, relationships between domains and entities (eg, drug or condition), classifications of entities, WA intents (target of a user’s query), and metadata elements. The ontology underpinning WA contains the domains of knowledge found in Micromedex’s Quick Answers database, drug–drug interactions and IV compatibility.

Connecting Micromedex’s HIDS (Figure 1, green) to IBM cloud applications (Figure 1, blue) is the representational state transfer application interface (REST API) that maintains interoperability and transfer of data between system components. The Micromedex UI, cloud applications interface, and WA are supported by the conversation manager, based on rules for business logic which establish system awareness of clinical reference content, allowing a query to be interpreted by WA. WA is part of the UI that links user queries to information contained in the Micromedex knowledge base. Other operations found within IBM cloud applications include internal functions for system maintenance controlled from the IBM cloud, as well as a test UI to evaluate new codes and interfaces as they are developed. The updater service handles daily content updates and deployment of all client and server applications that interface between WA and cloud DB2. The summary drug information used by WA is stored in the DB2 database. The DB2 database links WA, the ontology, and the CMS, handling tracking and logging of user queries for quality review and ongoing WA system training.

Intents are the intended target of a user’s query. For example, user may desire to determine adverse effects of a drug and thus, there is an intent named “adverse effects.” An entity represents a drug or condition relevant to a specific intent and provides context for that intent. Intents are represented hierarchically within the ontology. Intents such as “pediatric dosing,” “FDA and non-FDA uses,” “dose adjustments,” “administration,” “contraindications,” “precautions,” “adverse effects,” “black box warnings,” “risk evaluation,” and “mitigation strategy,” “drug interactions,” “pregnancy and lactation,” “monitoring,” “mechanism of action,” “pharmacokinetics,” and “how supplied” are parent nodes in the ontology; “common side effects” and “serious side effects” are examples of child nodes under the parent node of “adverse effects.”

WA’s NLP, ML, and integrated ontology allows WA to retrieve answers to common medication-related questions by matching queries to underlying intents. WA’s natural language engine interprets a user’s query over its domain of drug knowledge, which assists the conversation service in identification of intents and entities.

## SYSTEM INTERFACES

### Keyword-based and WA-assisted searches

**Figure 2.**
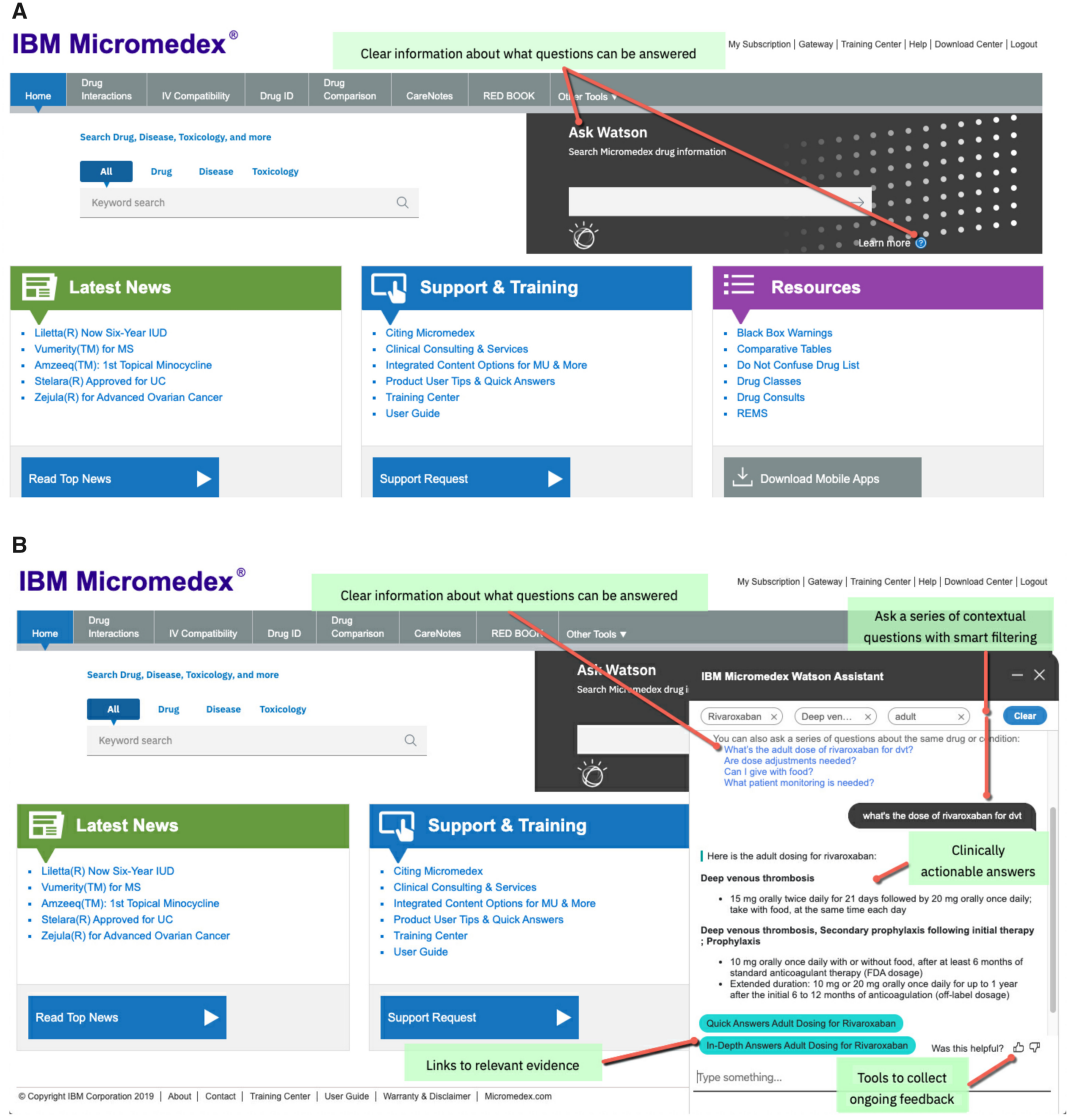
User interface. (A) Micromedex home page. Red arrow to “learn more” under “Ask Watson” explains what information Watson can provide (light green box), including examples of real-world questions, the types of information Micromedex with Watson understands (ie, drug information, drug interactions, and IV compatibility) and what Micromedex with Watson does not understand (ie, in-depth answers, NeoFax/pediatrics, toxicology, diseases, laboratory, alternative medicine, reproductive risks and third party content such as Martindale and Index Nominum). (B) WA chat window key features.

The information contained in Micromedex is accessible to users through the keyword search function available in Micromedex on the upper left of the Micromedex home screen (Figure 2A, upper left). It allows users to navigate to both detailed and summary level information, including related evidence and the ranking of that evidence. For example, a user searching for serious immunologic side effects of trastuzumab-qyyp can enter trastuzumab-qyyp in the keyword search bar and view “Adverse Effects” followed by “Serious” and “Immunologic” under that heading.

Alternatively, users can obtain information through WA’s natural language conversational search by directly entering questions using natural language into the “Ask Watson” search bar found on the upper right of the Micromedex home page (Figure 2a, black box). For example, a user can enter “What are serious immunologic side effects of Trazimera?” (Trazimera, trastuzumab-qyyp) in the “Ask Watson” search bar. WA opens a chat window and returns information specific to severe immunologic side effects of the drug (with links to further information) within the same chat window.

If WA requires more information to answer a query, it asks the user a question or series of questions to help WA understand the context and intent of the query, using smart filtering which helps the system resolve ambiguities. Smart filtering allows the conversational agent to ask for additional information when, for example, an entity match exists but the intent is unclear to WA. NLP maps entities and relationships related to the query to WA’s ontology to identify the intent of a query.^20^ Upon matching a query to intent, WA provides clinically actionable answers with evidence links within the chat window, drawing upon the same information and database that is interrogated by a keyword search of Micromedex.

### Conversational search

The conversational search allows clinicians to pose a series of questions on the same topic or on different topics. For example, a user can initiate a conversation with Watson by entering “What’s the adult dose of rivaroxaban for DVT?” (DVT, deep vein thrombosis; Figure 2B, right). In the conversation window, WA provides the adult dosing information for rivaroxaban with links to either “Quick Answers” or “In-Depth Answers” for rivaroxaban. When asking a series of questions, WA uses smart filtering to provide answers that include the context of prior questions by maintaining the identified entities over the course of the conversation. For example, a user can append “Are dose adjustments needed?” to the conversation without repeating the drug name. WA then returns “Here are the rivaroxaban dose adjustments,” followed by dose adjustments for renal impairment, hepatic impairment, geriatrics, bariatric surgery, and obesity, along with links to more information. If the user changes the topic of the conversation to another drug, WA will overwrite previous drugs and conditions and answer based on the new drug or condition. When WA cannot map a query to an intent, for example, when a user enters “off-label uses of erdafitinib,” WA responds with “I did not find adult non-FDA uses for erdafitinib.”

## MATERIALS AND METHODS

### System development and training

#### Ground truth

An initial ground truth was generated by clinical subject matter experts (SMEs), clinical content technical specialists, IBM research professionals, clinical pharmacists, and pharmacy students. This ground truth included common questions and frequently accessed information in Micromedex, mapped to correct intents and answers as determined by SMEs. To further development of the ground truth, a preliminary concept map of common questions was developed that linked questions to information contained in the Micromedex Drug Information knowledge base.

#### NLP training

A supervised learning approach was used to train the NLP components native to WA. The NLP system components disambiguated utterances (questions) by parsing named entities, such as medications, treatments, and conditions, mapping the information to entities modeled in the ontology. The ground truth provided the labeled training data that informed the mapping of system outputs. System inputs are a combination of the original concept map and real-world examples sourced from WA users. Corresponding answers (output) are drawn from Micromedex’s Quick Answer content, which is created and maintained by CMS clinical editors and by lexicon content specialists, updated daily in Micromedex, and supported by corresponding monographic content and references from clinical studies.

#### Machine learning components

The ML components integrated with WA include methods to predict and classify information and data. Specifically, an ML approach using support vector machines (SVM)^21–24^ is used to optimize solutions when users require additional suggestions. Although use of neural networks and other deep learning approaches were becoming more commonplace during development of Watson, the SVM approach used by WA remains one of the most robust methods available to perform the core classification task of matching utterances to intents when only small training sets are available. During system training, the SVM refined suggestions to end-users with inputs that comprised both the syntactic and semantic features obtained from the NLP components that parsed and classified entities and intents from user queries. System outputs were further refined within user queries to optimize search results. The training examples and the SVM native to WA are responsible for the deductive aspects of WA that allow it to function as a semantic reasoner.

#### System optimization

The native WA system—integrated with Micromedex—underwent successive rounds of iterative training using labeled examples curated from user utterances that were mapped to intents. Questions posed by pharmacists, nurses, physicians, clinicians, and medical librarians were used for system training during further refinement of the ground truth. Intents and real-user utterances were iteratively added. After each system enhancement, information from chat logs and other user feedback are systematically analyzed to uncover information used to fine-tune the system.

Utterances that return unexpected results are reviewed by clinical pharmacists and project developers to determine underlying intents. Results such as these are used to improve system training and performance and are integrated into the ground truth of the system. SMEs periodically review random chat log entries to examine the clinical output, identify defects, review which intent was fired given each utterance, and confirm the clinical accuracy of system-generated matches, informing ongoing system training and enhancements.

### Frequency of intents and accuracy of matching

Intents triggered by users during the initial 4-month use of WA at a mid-size (300 bed) tertiary care facility were recorded. Users were primarily pharmacists, nurses, and other clinical staff. Utterances were grouped by intent type to which they were mapped by WA, and frequency of intents was tabulated. WA uses a proprietary classification and detection method using SVMs to optimize features and match utterances to intents.

To examine accuracy of matching queries to intents, 400 sequential user queries collected over a 2-week period were analyzed. Each query was independently reviewed by 2 clinical pharmacists (SMEs). Each SME was blinded to intents assigned by the other SME and by WA. Any disagreements between the first 2 SMEs were arbitrated by a third SME who was also blinded to intents assigned by SMEs and WA. Any additional discrepancies were finalized using a consensus review to generate a final intent. The WA-assigned intents were compared to the ground-truth intents established by SMEs, and the percent of intents correctly assigned by WA was calculated. The beyond-chance agreement between reviewers involved in establishing the ground truth for matching was measured with Cohen’s kappa.

To examine potential differences in users’ approaches to finding information using a standard keyword search versus a conversational agent, we compared the top intents triggered by WA users to analogous drug topics accessed by keyword searches of Micromedex over the course of 9 months. The number of intents or drug topics accessed through WA-assisted versus keyword search was calculated as a percent of total searches by each method. The top intents triggered in WA were compared to the frequency of analogous drug topics accessed through keyword searches of Micromedex.

## RESULTS

### Intents triggered

**Figure 3.**
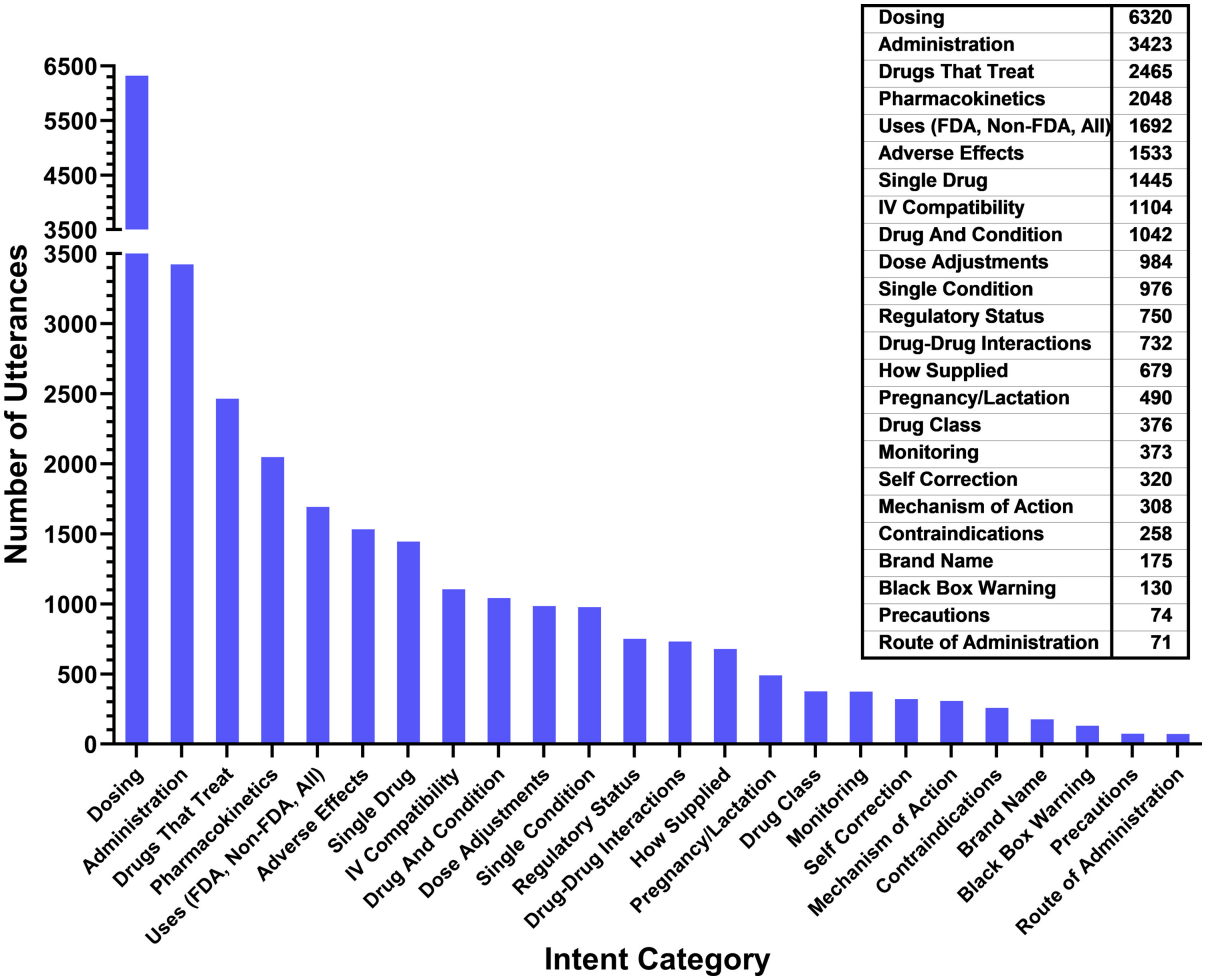
Frequency of intents. Utterances that mapped to queries (42 771 total queries) collected between October of 2018 through January 2019, grouped by category or intent to which it was mapped by WA.

There was a total of 42 771 conversations logged over the 4-month initial use study in late 2018; 27 768 (64.9%) of these conversations were mapped by WA to an intent, as determined by the system (Figure 3). Questions in the conversation logs ranged from very specific (ie, “What is the *t*_1/2_ of itraconazole?”) to general (“Heart attack”) to nonsensical (“dsfsdfsdfsdfysdftysdtfy…”). Representative user queries and intents to which they were mapped by the system are shown in Table 1. Unassigned queries are those that either lack the requisite information needed to map to an intent (ambiguous queries) or the information is unavailable in the Micromedex Quick Answers database (unavailable). Unassigned queries include those such as “Is there a term for increase in white blood cells” (no condition name was provided). Examples of unavailable queries include utterances such as “How to pronounce Lunesta” and “is magnesium phosphate available in South Africa,” as well as questions such as “what is 2 + 2” or “how do I print this.”

**Table 1.** Examples of user queries and intents (as determined by SME)

Example query	Intent
“What is the dose of adalimumab in plaque psoriasis?”	Dosing
“How fast can I run phenytoin sodium?”	Administration
“What drugs that treat migraine?”	Drugs that treat
“What drugs are used for asthma?”	
“What is the *t*_1/2_ of itraconazole?”	Pharmacokinetics
“FDA uses for Harvoni?”	Uses (FDA, non-FDA, all)
“Off label uses of secukinumab?”	
“What is dronedarone used for?”	
“Show me serious adverse effects for cyclosporine”	Adverse effects
“Tacrolimus”	Single drug^a^
“Are amiodarone and cimetidine iv compatible?”	IV compatibility
“Amoxicillin anthrax”	Drug and condition^b^
“Dose adjustments for gentamicin”	Dose adjustments
“Heart attack”	Single condition^c^
“What schedule is lorazepam?”	Regulatory status
“What are the drug interactions for atorvastatin?”	Drug–drug interactions
“Does prednisone come in capsules?”	How supplied
“How supplied for diltiazem?”	
“What is the pregnancy category of captopril?”	Pregnancy/lactation
“Is abraxane safe in breastfeeding?”	
“zovirax class”	Drug class
“What to monitor for carbamazepine?”	Monitoring
Queries vary	Self-correction
“Show me mechanism of action of droperidol”	Mechanism of action
“Contraindications of mesoridazine”	Contraindications
“Does morphine have black box warnings?”	Black box warning
“Precautions for trintellix”	Precautions
“By what route is naloxone given?”	Route of administration
“What is the brand name of doxazosin?”	Brand name
“What is the drug class of amphotericin b?”	Drug class

^a^ Watson returns use information.

^b^ Watson currently returns drug and condition but will eventually return a choice of adverse effects, precautions, contraindications, uses, or dosing.

^c^ Watson returns drugs used for named condition.

### Frequency and accuracy of intents

The frequency of intents that was triggered by queries during the initial 4-month study period is shown in Figure 3. Most questions that were mapped to an intent by WA involved dosing and administration, followed by drugs that treat (certain conditions), pharmacokinetics, and on- and off-label usage.

The accuracy of WA intents assigned to 400 sequential queries, regardless of availability in Micromedex, was 61.75%, as determined by SMEs displaying substantial inter-rater agreement (Cohen’s kappa 0.71, 95% CI 0.66–0.76).^25–27^ Of these 400 queries, 38.25% (*N* = 153) were not successfully matched to an intent, for reasons as follows: 14.25% (*N* = 57) were incorrectly mapped by WA; 13.5% (*N* = 54) were unavailable in WA; 5.25% (*N* = 21) were too ambiguous to determine accuracy, and 5.25% (*N* = 21) contained typographic errors or abbreviations that were not recognized by WA.

Mismatches of queries to intents by WA (*N* = 57) that SMEs considered to be within WA’s domain of knowledge included: IV compatibility, 13; drug–drug interactions, 6; drug dosage for condition, 5; drug case, 5; drugs that treat (specific conditions), 4; precautions, 3; pharmacokinetics, 2; administration of drug, 1; brand name, 1; condition case, 1; mechanism of action, 1; monitoring of drug, 1; pregnancy and lactation, 1; and regulatory status, 1. In addition, WA returned intents for 12 queries that should have returned “unavailable.” The accuracy analysis identified 112 queries (28% of 400) that were unanswerable by WA. These included queries that were either ambiguous, contained unrecognizable typographical errors, or related to content unavailable in the Micromedex Quick Answers database. Of the 288 queries that SMEs determined were within WA’s knowledge domain and specific enough to be answered by WA, 231 (80% of 288 answerable queries) were determined by SMEs to be mapped to the correct intent.

The accuracy analysis triggered all of the intents shown in Figure 3, as well as queries related to “care notes,” “comparative efficacy,” “do not crush consult,” “drug consult,” “drug–alcohol interactions,” “drug food interactions,” “drugs that cause,” “Neofax,” and “Redbook.” Training for these terms and phrases was added after the initial use data collection to facilitate linkage of these phrases to appropriate intents.

### WA-assisted search versus keyword search

A total of 126 765 conversations with WA were mapped to intents during a 9-month study period. The most common topic accessed using WA was dosing, followed by administration, drugs that treat (specified conditions), uses (including both FDA and non-FDA), pharmacokinetics, IV compatibility, and adverse effects. Although dosing was also the most common drug topic accessed in a keyword search of Micromedex, the frequency of searches differed by search method (dosing, 23.9% with WA vs 43.3% with keyword search); other search topics and frequencies also differed according to search method (Table 2).

**Table 2.** Top 7 topics accessed with WA search versus keyword search

Watson assistant intent	WA search, *N* (%)	Keyword search, *N* (%)	Watson assistant example query
Dosing	30 239 (23.9%)	34 322 058 (43.3%)	“What is the dosage of azithromycin?”
Administration	14 520 (11.5%)	2 764 488 (3.5%)	“Can burosumab be administered SQ in the legs or abdomen?”
Drugs that treat^a^	10 199 (8.0%)	190 217 (0.2%)	“Medications used for hypertension”
Uses (FDA, non-FDA, all)	8651 (6.8%)	5 641 662 (11.5%)^a^	“Can moxifloxacin be used in UTI?”
Pharmacokinetics	8495 (6.7%)	1 544 830 (1.9%)	“How is nimbex metabolized?”
IV compatibility	7942 (6.3%)	435 461 (0.05%)	“Is daptomycin compatible with micafungin”
Adverse effects	6676 (5.3%)	5 416 938 (6.8%)	“Diazepam side effect”

^a^ Sum of on-label and off-label uses.

## DISCUSSION

This report describes an AI-enabled conversational agent, WA, linked to a pharmacological database, Micromedex, to identify answers to clinicians’ medication-related questions posed using natural language. In this study, 62% of all natural language queries were correctly matched to intent by WA, and 80% of queries within WA’s domain of knowledge were correctly matched by WA, according to a gold standard developed by 2 independent SMEs.

WA relies on an ontology and NLP to map queries to intents, as compared to keyword searches related to drug topics. The content most frequently accessed by either search method was related to dosing, but the proportion differed by method. Although dosing made up almost half of all topics accessed by keyword search, only about a quarter of intents were linked to dosing using WA. There were 3 times as many queries related to medication administration using WA as compared to keyword search; other topics and intents and their frequencies also differed by search method. These differences may reflect the ability of WA to aid users in articulating questions that are not easily answered with a standard keyword search. Further studies are underway which explore the user experiences associated with each method.

Many queries were either too vague to be matched to an intent or were related to information not contained in WA’s knowledge domain. In the case of vague queries, WA provides users with a choice of content areas for further consideration to facilitate the matching process. Many of the queries that were either not mapped to an intent or not mapped correctly were related to misspellings or unrecognized abbreviations. During the accuracy analysis, SMEs were able to map some of these to appropriate content, but many could not be interpreted. To assist with this problem, WA’s ‘fuzzy match’ feature can help correct minor misspellings; however, it can also fail to recognize a misspelling, replace a misspelled word with a word may then be incorrectly matched to an intent, or identify a correctly spelled word as a misspelled word. Furthermore, highlighting the types of information that can and cannot be accessed in WA would likely reduce the number of queries related to information not available in WA.

An important measure of system performance is whether a user’s question was answered appropriately. To determine this, a ground truth for accuracy of intent mapping was developed by SMEs with substantial inter-rater agreement. The accuracy of intent mapping by WA for all queries was 61.75%. Two-thirds of the queries not successfully mapped were either unavailable in WA’s knowledge domain or too ambiguous to map. Some of the utterances that WA was unable to assign to an intent contained phrases such as “other names,” “how often,” “taken together,” “ran together,” “mix,” “IV…hang,” and “absorption.” For queries that were mapped incorrectly by WA, the greatest number were related to IV compatibility, followed by those that should have been categorized as unavailable in WA but returned an intent. Many of the incorrectly mapped queries were due to the system’s difficulty with identification of contextual features, as well as difficulty with making inferences from natural language.

There are few rigorous studies of conversational agents in healthcare, and none related to pharmacy.^28^ Thus, the current study is the first to report the technical performance of a conversational agent aimed at answering medication-related questions using ground-truth intents established by 2 independent SMEs, underpinned by a curated knowledge base.

This work has several limitations. First, the study involved data collected over a relatively short span of time, and only a small subset of queries were independently evaluated by 2 SMEs. A more comprehensive review of system-generated matches is underway, with results informing future system enhancements. Second, this work describes early adopters’ experiences with WA and may not reflect the types of queries or system performance after users gain experience with the tool or after optional integration of the tool into an electronic health record.

## CONCLUSIONS

We have described the architecture and early user experiences with WA, an AI-based conversational agent linked to a curated pharmacological knowledge base. WA allowed users to access a broad range of topics and correctly linked most user queries to intents. One-third of queries not mapped were either ambiguous or related to information not available to WA. The distribution of information sought via WA’s conversational search versus keyword search differed depending on search method; WA may enable users to articulate different types of questions than what is sought using a standard keyword search of Micromedex.

## FUNDING

This work was funded by IBM.

## AUTHOR CONTRIBUTIONS

AMP, BS, AB, JH, and GPJ contributed to project design and drafting of the manuscript. MB, JH, and AB contributed to data acquisition; AMP and SW performed data analysis. AMP, NK, BB, and GJP provided leadership for the project. All authors contributed to data interpretation, manuscript revision, and approval of the final manuscript.

## CONFLICT OF INTEREST STATEMENT

The authors are employed by IBM Watson Health.
